# Skeletal Muscle Growth Hormone Receptor Signaling Regulates Basal, but Not Fasting-Induced, Lipid Oxidation

**DOI:** 10.1371/journal.pone.0044777

**Published:** 2012-09-14

**Authors:** Archana Vijayakumar, YingJie Wu, Nicholas J. Buffin, Xiaosong Li, Hui Sun, Ronald E. Gordon, Shoshana Yakar, Derek LeRoith

**Affiliations:** 1 Division of Endocrinology, Diabetes, and Bone Diseases, Department of Medicine, Mount Sinai School of Medicine, New York, New York, United States of America; 2 David B. Kriser Dental Center, Department of Basic Science and Craniofacial Biology, New York University College of Dentistry; 3 Departments of Medicine and Neuroscience, Albert Einstein College of Medicine, New York, New York, United States of America; 4 Department of Pathology, Mount Sinai School of Medicine, New York, New York, United States of America; University of Cordoba, Spain

## Abstract

**Background:**

Growth hormone (GH) stimulates whole-body lipid oxidation, but its regulation of muscle lipid oxidation is not clearly defined. Mice with a skeletal muscle-specific knockout of the GH receptor (mGHRKO model) are protected from high fat diet (HFD)–induced insulin resistance and display increased whole-body carbohydrate utilization. In this study we used the mGRHKO mice to investigate the role of muscle GHR signaling on lipid oxidation under regular chow (RC)- and HFD- fed conditions, and in response to fasting.

**Methodology/Principal Findings:**

Expression of lipid oxidation genes was analyzed by real-time PCR in the muscles of RC- and HFD- fed mice, and after 24 h fasting in the HFD-fed mice. Expression of lipid oxidation genes was lower in the muscles of the mGHRKO mice relative to the controls, irrespective of diet. However, in response to 24 h fasting, the HFD-fed mGHRKO mice displayed up-regulation of lipid oxidation genes similar to the fasted controls. When subjected to treadmill running challenge, the HFD-fed mGHRKO mice demonstrated increased whole-body lipid utilization. Additionally, under fasted conditions, the adipose tissue of the mGHRKO mice displayed increased lipolysis as compared to both the fed mGHRKO as well as the fasted control mice.

**Conclusions/Significance:**

Our data show that muscle GHR signaling regulates basal lipid oxidation, but not the induction of lipid oxidation in response to fasting. We further demonstrate that muscle GHR signaling is involved in muscle-adipose tissue cross-talk; however the mechanisms mediating this remain to be elucidated.

## Introduction

The metabolic outcomes associated with enhanced lipid oxidation in the skeletal muscle are contradictory. On the one hand, activation of lipid oxidation during and following exercise or fasting is associated with beneficial metabolic effects such as enhanced mitochondrial biogenesis and respiration, reduced inflammation, and improved glucose utilization. On the other hand, obesity and insulin resistance are also associated with increased lipid oxidation but result in impaired insulin action in the skeletal muscle (reviewed in [1]). The reason for this dichotomy was explained by Koves *et al* who demonstrated that insulin resistant muscles have lost their metabolic flexibility or the ability to switch between using carbohydrates and lipids in the fed and fasted states, respectively. They further reported increased fatty acid (FA) delivery and a higher rate of incomplete lipid oxidation in the muscles of obese, insulin resistant mice [2]. The ability of enhanced lipid utilization to hamper cellular glucose uptake and utilization has been described as the “glucose-fatty acid” cycle and may be involved in the above-mentioned effects [3], [4].

Growth hormone (GH) is known to enhance whole-body lipid oxidation; however its regulation of substrate preference specifically in the skeletal muscle is not clearly defined [5], [6], [7]. We have previously reported that mice with genetic loss of growth hormone receptors (GHR) specifically in the skeletal muscle (mGHRKO model) are protected from high-fat diet (HFD)-induced obesity and insulin resistance. Despite having a similar food intake, the mGHRKO mice had reduced fat mass, improved whole-body insulin sensitivity, and increased energy expenditure compared to the control mice when subjected to HFD feeding. Hyperinsulinemic-euglycemic clamp studies revealed higher insulin-stimulated glucose uptake in the skeletal muscle and adipose tissue of the HFD-fed mGHRKO mice. Moreover, the respiratory exchange ratio (RER), which is an indicator of substrate preference, was also higher in the HFD-fed mGHRKO mice in the light phase, indicative of enhanced carbohydrate utilization [8]. Thus, using the mGHRKO mice we sought to analyze the effect of muscle GHR signaling on lipid oxidation, and the ability of the muscle to switch between fuel sources in the fed-to-fasted transition.

PGC1α (peroxisome proliferator-activated receptor γ coactivator 1-α) is a master regulator of lipid metabolism and mitochondrial function in the skeletal muscle, and is induced in response to cues of increased energy demand such as fasting, and exercise (reviewed in [9]). The importance of PGC1α in mediating the metabolic benefits of exercise is evident from the skeletal muscle-specific PGC1α knockout mice that demonstrate decreased locomotor activity as well as a lower threshold for endurance exercise, while the opposite is observed in PGC1α transgenic mice [10], [11]. PGC1α is subject to transcriptional as well as post-translational regulation. One important post-translational modification of PGC1α that increases its activity is deacetylation, and the NAD^+^-dependent histone deacetylase sirtuin1 (SIRT1) has been implicated in this process [12]. In the muscle, PGC1α is involved in lipid uptake and oxidation, mitochondrial biogenesis and oxidative phosphorylation, glucose uptake, and fiber-type specification. These diverse effects are mediated by the association of PGC1α with various transcription factors such as the peroxisome proliferator activated receptors (PPARs), estrogen- related receptors (ERRs), nuclear respiratory factor (NRF), and myocyte-specific enhancer factor 2C (MEF2C). In addition to increasing their transcriptional activity, PGC1α has also been shown to induce the expression of some of these transcription factors (reviewed in [13]). Thus, the gene expression signature of transcription factors associated with PGC1α is a good indicator of the state of activation of lipid oxidation pathways, and we applied this to our present study.

Our data ascribe a role for skeletal muscle GHR signaling in regulating basal lipid oxidation, but not in response to fasting. The mGHRKO mice demonstrate down-regulation of genes involved in PGC1α-dependent lipid oxidation in the muscle under both regular chow (RC)- and HFD- fed conditions, suggesting a direct role of GHR signaling in mediating muscle lipid oxidation. However, under states of increased energy demand such as fasting and exercise the HFD-fed mGHRKO mice are able to effectively activate a program of lipid oxidation similar to the HFD-fed control mice. Furthermore, we also show that the adipose tissue of the HFD-fed mGHRKO mice demonstrates increased lipolysis and free fatty acid (FFA) secretion under fasted conditions, suggesting a metabolic adaptation that increases FA supply to the muscle.

## Results

### mGHRKO Mice Demonstrate Down-regulation of Components of the PGC1α-dependent Lipid Oxidation Pathway in the Muscle

**Figure 1:**
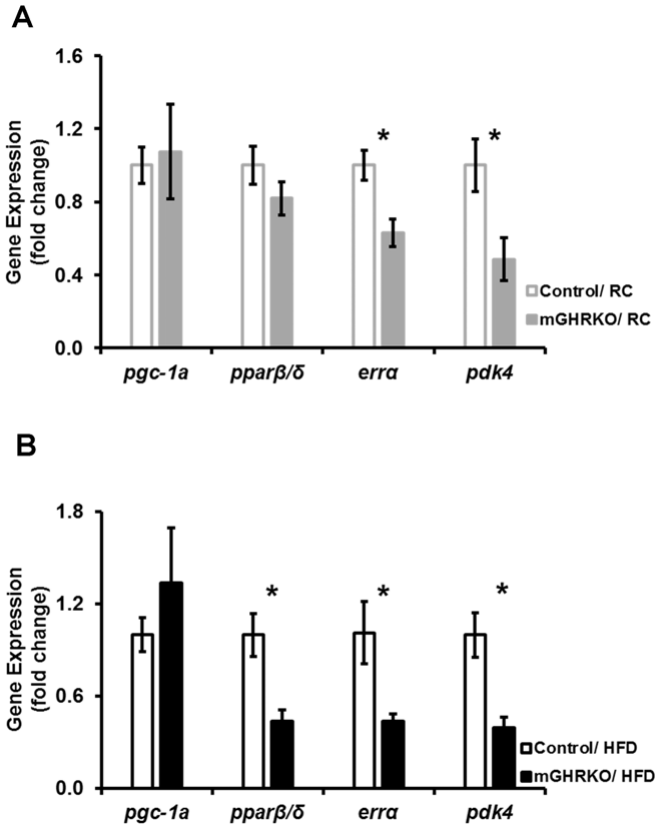
The muscles of mGHRKO mice display down-regulation of lipid oxidation genes. mRNA expression of indicated genes was determined by real-time PCR in quadriceps muscles of **A.** 16 wk-old regular chow (RC) -fed (n = 6 mice/genotype), and **B.** 14wk-high-fat diet (HFD) -fed (n = 14–16/group) control (open bars) and mGHRKO mice (closed bars). Each sample was probed three times and gene expression was normalized to *β-actin* (**A**) or *gapdh* (**B**) and represented as a fold change when compared to respective control (either RC- or HFD- fed). All values are represented as mean ± S.E.M. *−p≤0.05, One-way ANOVA.

Since PGC1α is implicated in activation of lipid oxidation pathways, we analyzed the expression of components of this pathway in the mGHRKO mice under RC- and HFD- fed conditions. While no difference in the expression of *pgc1α* and *pparβ/δ (peroxisome proliferator- activated receptor β/δ)* was observed in the muscles of 16wk-old RC-fed mGHRKO mice when compared to controls, the expression of *errα (estrogen-related receptor α)* and *pdk4 (pyruvate dehydrogenase kinase 4)* were significantly reduced in the mGHRKO mice relative to the control mice **[** **Figure 1A** **]**. However, under HFD-fed conditions, the expression of *pparβ/δ*, *errα*, and *pdk4* were significantly down-regulated in the mGHRKO mice compared to the control mice, despite similar expression of *pgc1α* **[** **Figure 1B** **]**. These data suggest that the lipid oxidation is suppressed in the skeletal muscle of the mGHRKO mice under RC- as well as HFD- fed conditions.

### Muscle Mitochondrial Content in the mGHRKO Mice is Similar to the Controls

**Figure 2:**
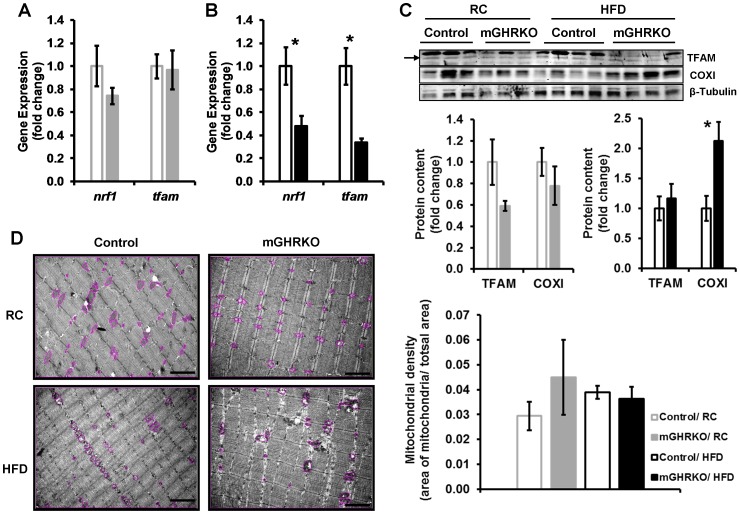
Mitochondrial content is not altered in the muscle of the HFD-fed mGHRKO mice. mRNA expression of *nrf1* and *tfam* were determined by real-time PCR in quadriceps muscles of **A.** 16 wk-old RC-fed (n = 6 mice/genotype), and **B.** 14wk HFD-fed (n = 14–16/group) control and mGHRKO mice. Each sample was probed three times and gene expression was normalized to *β- actin* (**A**) or *gapdh* (**B**) and represented as a fold change when compared to respective control (either RC- or HFD- fed). **C.** Protein content of TFAM and COXI were determined in protein lysates isolated from quadriceps muscle of RC- and HFD- fed control and mGHRKO mice (n = 3–4/group). β- tubulin was used to determine equal loading (top). The protein content was normalized to that of β- tubulin, and represented as a fold change compared to respective control (either RC- or HFD- fed) (bottom). Arrow indicates appropriate TFAM band. **D.** Electron micrographs of quadriceps muscle of 16wk-old or 14wk-HFD-fed mice (n = 3–5/group; representative images are shown). The mitochondria in the section are encircled. Scale bar represents 2 µm (left). The area of the mitochondria was quantified using ImageJ software and mitochondrial density was determined as a ratio of the total area of the mitochondria to the total area of the field (right). All values are represented as mean ± S.E.M. *- p≤0.05, One-way ANOVA.

Muscle fibers can be categorized as oxidative and glycolytic fibers based on their fuel source, that is, triglycerides and glycogen respectively. PGC1α has been shown to enhance muscle oxidative capacity by promoting mitochondrial biogenesis via activation of the transcription factor NRF1 (nuclear respiratory factor 1) and its downstream target, mitochondrial transcription factor A (TFAM). The expression of *nrf1 and tfam* was not altered in the quadriceps of 16 week old RC-fed mGHRKO mice **[** **Figure 2A** **]**, but was significantly decreased in that of the HFD-fed mGHRKO mice relative to the controls **[** **Figure 2B** **]**. This was associated with 40% reduction or unchanged TFAM protein content in the RC- and HFD- fed mGHRKO mice respectively **[** **Figure 2C** **]**.

Muscle fiber cross-sectional area was significantly lower in the mGHRKO mice under RC-fed conditions, but there was no difference between the genotypes under HFD-fed conditions **[Figure S1A, S1C]**. The expression of the different myosin heavy chain (MHC) isoforms is indicative of fiber type with progression from oxidative to glycolytic fibers entailing a switch in expression of the isoforms from MHCI>MCHIIa>MHCIIx>MHCIIb respectively. The expression of *mhcI* and *mhcIIa*, which are expressed in oxidative fibers, in the quadriceps was similar between the control and mGHRKO mice under both RC- and HFD- fed conditions **[Figure S1B, S1D]**. The protein expression of mitochondrial-encoded cytochrome oxidase subunit I (COXI) was either not altered or significantly increased in the quadriceps of the mGHRKO mice relative to controls under RC- and HFD- fed conditions respectively **[** **Figure 2C** **]**. Further, mitochondrial density was also not different between the control and mGHRKO mice under both RC- and HFD- fed conditions **[** **Figure 2D** **]**. Also, staining of gastrocnemius muscles for cytochrome oxidase activity also did not reveal any significant differences between the genotypes under HFD-fed conditions **[Figure S1E]**. However, the HFD-fed mGHRKO mice displayed significantly higher expression of *mhcIIb* which is suggestive of greater glycolytic capacity **[Figure S1D]**.

These data suggest that the loss of GHR signaling in the skeletal muscle did not affect mitochondrial capacity of the muscle under RC- and HFD- fed conditions and also indicate the existence of PGC1α-independent pathways regulating mitochondrial DNA (mtDNA) replication and transcription, as well as, muscle fiber type as will be discussed later.

### HFD-fed mGHRKO Mice Display Induction of Lipid Oxidation Genes in Response to Fasting

**Figure 3:**
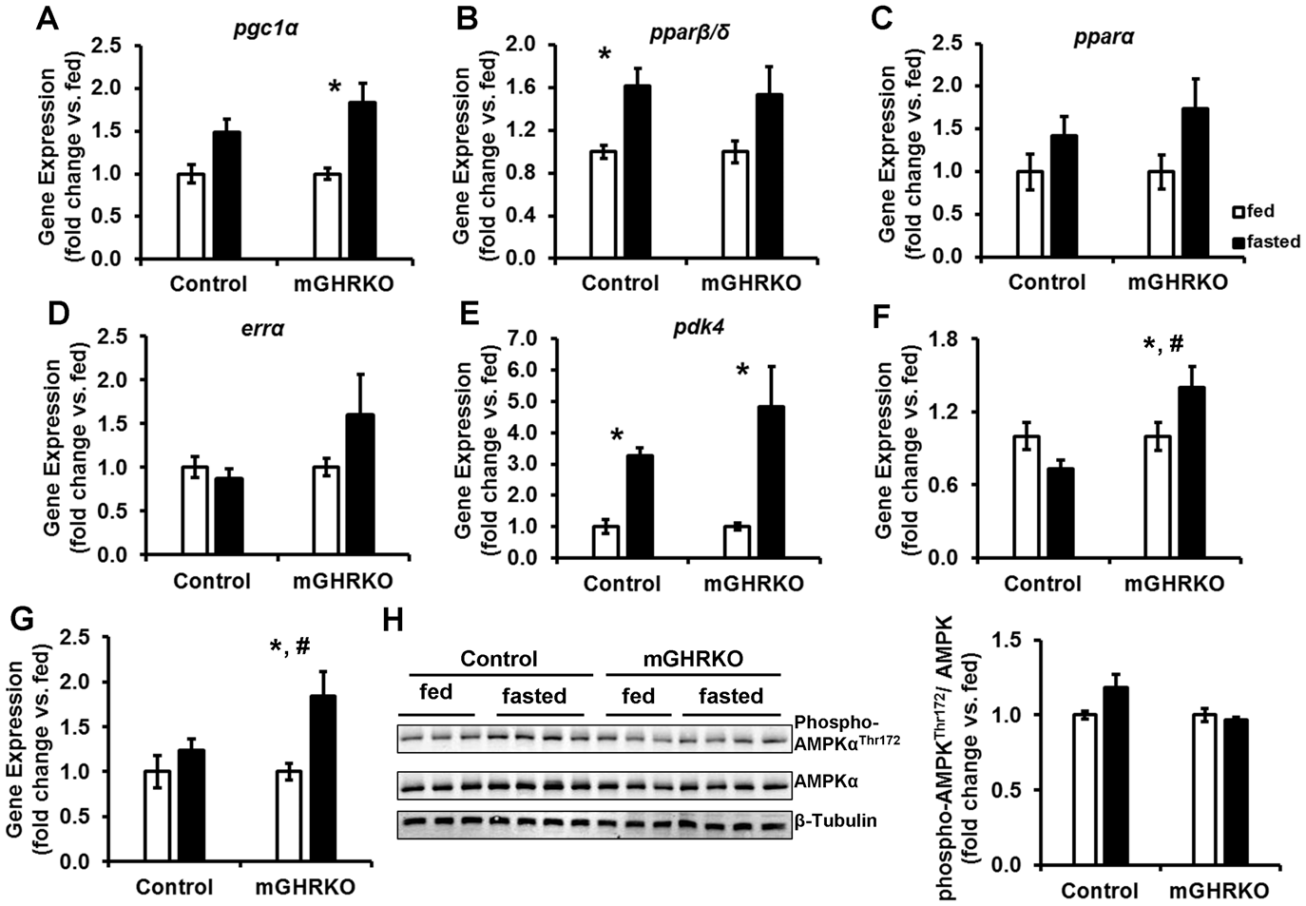
Activation of lipid utilizing pathways in muscles of HFD-fed control and mGHRKO mice in response to 24 h fasting challenge. 14-wk HFD-fed control and mGHRKO mice were subjected to a 24 h fasting challenge. (**A–G**)**.** mRNA expression of indicated genes in quadriceps muscles of fed, and 24 h fasted mice (n = 6–8/group). Each sample was probed three times and gene expression was normalized to *β-actin* and represented as a fold change when compared to the fed mice for each genotype. **H.** Phosphorylation of AMPKα at Thr^172^ was determined in protein lysates isolated from quadriceps muscle of fed and fasted control and mGHRKO mice. Levels of phosphorylated AMPKα was normalized to total protein content of AMPKα and represented as a fold changed compared to fed mice of each genotype. β- tubulin was used to determine equal loading (n = 6–8/group; representative blots are provided). All values are represented as mean ± S.E.M. *−p≤0.05 versus respective fed group, #−p≤0.05 fasted control versus fasted mGHRKO, Two-way ANOVA.

Nutrient availability determines substrate preference; fasting promotes lipid utilization and activation of lipid oxidation pathways in a PGC1α-dependent manner. Thus, we subjected the HFD-fed mice to a 24 h fasting, and analyzed the induction of genes involved in the PGC1α-dependent lipid oxidation pathway. Fasting increased the expression of *pgc1α*, *pparβ/δ*, and *pparα* in both control and mGHRKO mice when compared to the fed mice of each genotype **[** **Figure 3A, 3B, 3C** **]**. The expression *errα* tended to be higher in the fasted mGHRKO mice relative to the fed mGHRKO mice, while no alteration in *errα* expression was seen in the fasted control mice **[** **Figure 3D** **]**. *pdk4* expression was significantly increased in both the fasted controls and mGHRKO mice **[** **Figure 3E** **]**. Fasting significantly increased the expression of *sirt3* in the muscles of the HFD-fed mGHRKO mice, and this was not evident in the fasted control mice **[** **Figure 3F** **]**. Interestingly, there was no difference in basal *sirt3* expression in the control and mGHRKO muscles in the fed state **[Figure S2]**.

PGC1α is activated by deacetylation and phosphorylation which are mediated by SIRT1 and AMP-activated protein kinase (AMPK). While there was no difference in the basal expression of *sirt1* in the fed state **[Figure S2]**, fasting was associated with a significant induction of *sirt1* only in the mGHRKO mice, but not the control mice **[** **Figure 3G** **]**. Further, there was no significant difference in AMPK phosphorylation upon fasting in both the genotypes **[** **Figure 3H** **]**. Thus, despite lower basal expression, the HFD-fed mGHRKO mice were able to up-regulate the expression of lipid oxidation genes in response to fasting.

### HFD-fed mGHRKO Mice Exhibit Increased Lipid Utilization during a Treadmill Running Challenge

**Figure 4:**
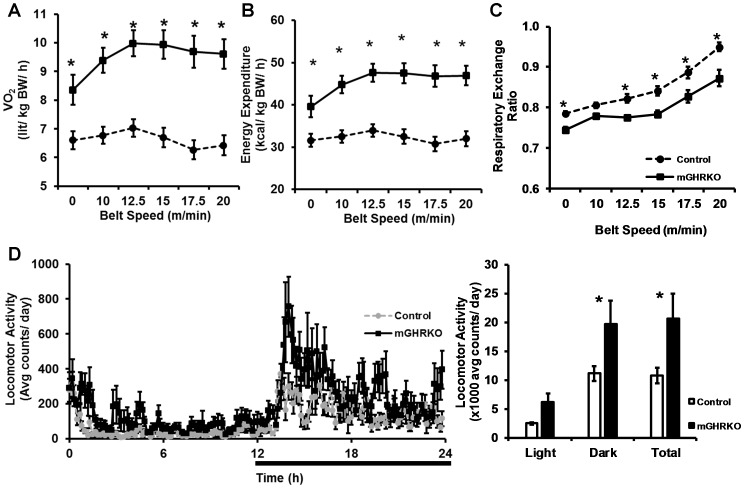
Increased lipid utilization during a treadmill running challenge and greater locomotor activity in the HFD-fed mGHRKO mice. 14wk-HFD-fed control and mGHRKO mice were subjected to a treadmill running study. **A.** Oxygen consumption (VO_2_), **B.** Energy expenditure after normalization to BW, and **C.** Respiratory exchange ratio were measured during the treadmill running study (n = 8/group). **D.** 24 h locomotor activity (left) was measured as beam breaks in the horizontal and vertical axes in 14wk-HFD-fed control and mGHRKO mice, and summarized as light phase, dark phase and total locomotor activity (right). Bar represents dark phase. All values are represented as mean ± S.E.M. *−p≤0.05, One-way (**D**) or Two-way (**A–C**) ANOVA.

Having observed activation of lipid oxidation genes in the muscles of the HFD-fed mGHRKO mice in response to fasting, we subjected the HFD-fed mice to a treadmill running challenge after two training sessions to stimulate lipid utilization. During the study, the HFD-fed mGHRKO mice displayed a significant increase in oxygen consumption (VO_2_) and energy expenditure, normalized to body weight, indicating that they have greater aerobic capacity than the control mice **[** **Figure 4A, 4B** **]**. Additionally, the mGHRKO mice demonstrated lower RER indicating their reliance on lipids as the predominant fuel source **[** **Figure 4C** **]**. We also measured 24 h locomotor activity and found that the HFD-fed mGHRKO had significantly greater dark phase locomotor activity when compared to the controls **[** **Figure 4D** **]**. Thus, the HFD- fed mGHRKO mice are able to activate lipid oxidation in response to exercise, perhaps to a greater extent than HFD- fed control mice.

### Adipose Tissue of HFD-fed mGHRKO Mice Demonstrates Increased Lipolysis under Fasted Conditions

**Figure 5:**
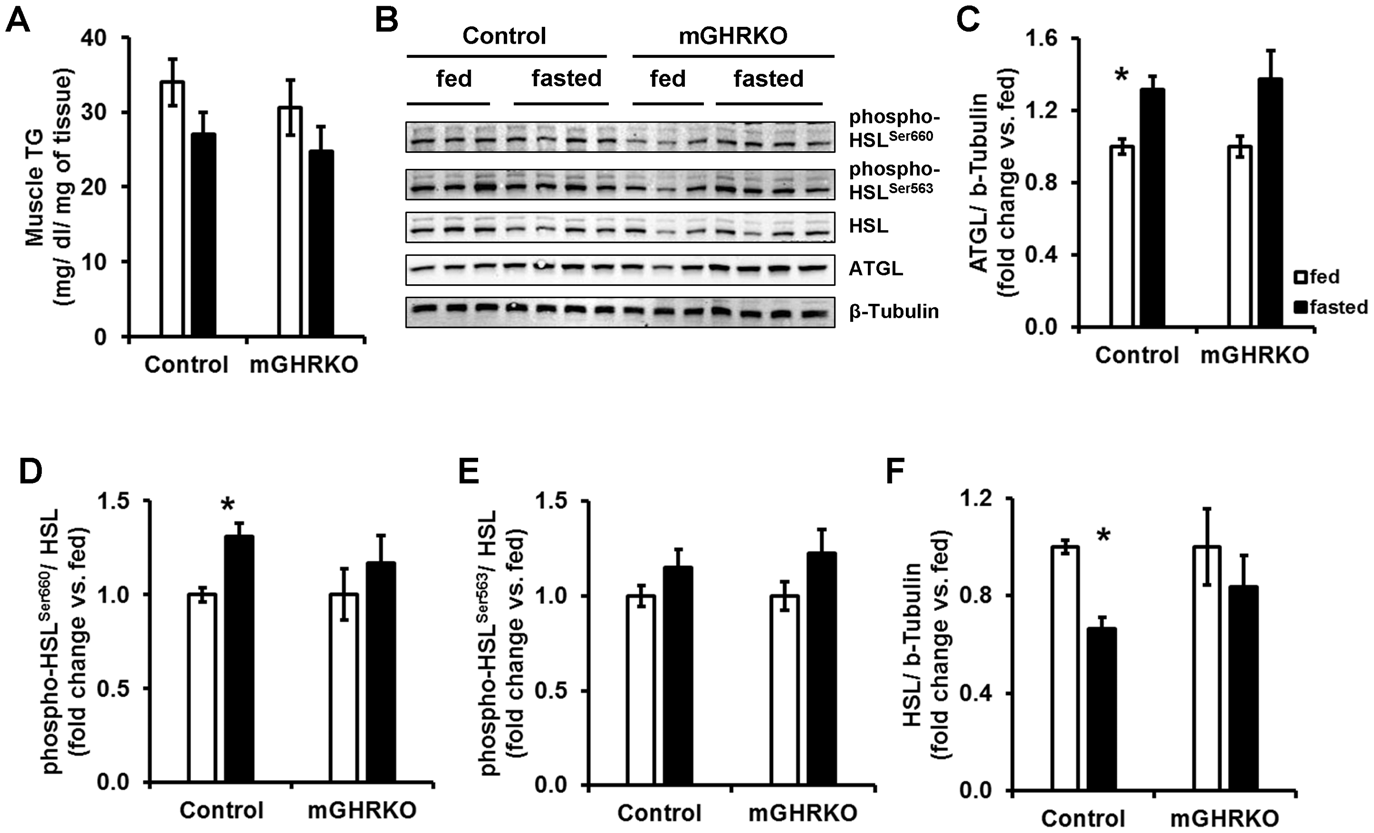
Lipid handling the control and mGHRKO mice in response to 24 h fasting. 14-wk HFD-fed control and mGHRKO mice were subjected to a 24 h fasting challenge. **A.** Muscle TG content in the gastrocnemius was determined in the fed and fasted mice (n = 6–8/group). (**B–F**). Western blots were performed to determine total protein content of ATGL and HSL, and the phosphorylated levels of HSL at residues Ser^660^ and Ser^563^ in the quadriceps muscle. β-tubulin was used to determine equal loading. Levels of phosphorylated HSL at Ser^660^ and Ser^563^ were normalized to total HSL content, while total protein content of ATGL and HSL were normalized to β-tubulin. The data are represented as a fold change compared to the fed state levels for each genotype (n = 6–8/group; representative blots are provided). All values are represented as mean ± S.E.M. *− p≤0.05 versus respective fed group, One-way ANOVA.

24 h fasting resulted in slightly lower intramuscular TG content in both the control and mGHRKO mice **[** **Figure 5A** **]**. This was further associated with a significant increase in the protein content of the triglyceride (TG) hydrolase, adipose triglyceride lipase (ATGL) in the muscles of the HFD-fed control mice but not the HFD-fed mGHRKO mice **[** **Figure 5B, 5C** **]**. We also looked at the activation status of the other important TG hydrolase, hormone sensitive lipase (HSL). The control, but not mGHRKO, mice demonstrated significantly higher phosphorylation of HSL at residue Ser^660^, but not Ser^563^ **[** **Figure 5B, 5D, 5E** **]**. Interestingly, fasting significantly decreased the total protein content of HSL in the control mice, but this was not observed in the mGHRKO mice **[** **Figure 5B, 5F** **]**. Further we detected a trend towards higher protein content of the FA transporter (FAT/CD36) in the skeletal muscle of fasted mGHRKO, but not control, mice relative to their fed counterparts (data not shown). These data suggest that fasting did not significantly induce lipolysis of intramuscular TG in the mGHRKO mice, and perhaps there was a greater influx of FA from the circulation.

**Figure 6:**
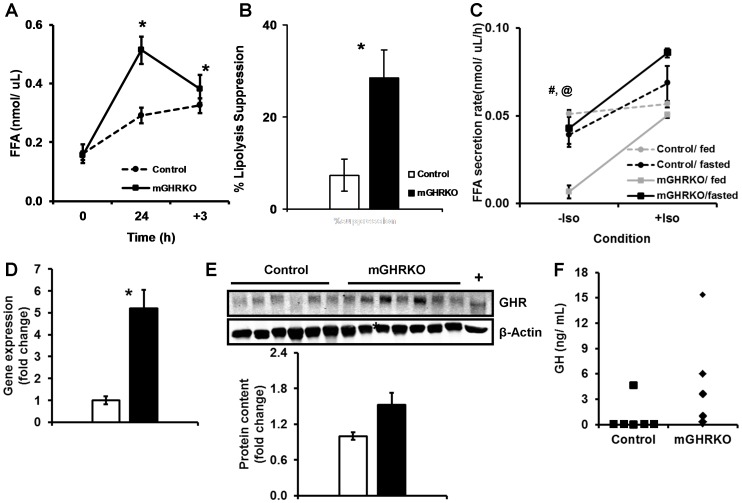
Adipose tissue of the fasted mGHRKO mice is more lipolytic than that of control mice. A. FFA levels were determined in the serum of 10–12-wk HFD-fed mice at baseline (0), after 24 h fasting (24), and after 3 h of re-feeding (+3). **B.** The suppression of lipolysis was determined as the percent change in FFA levels at the start and end of the re-feeding phase. **C.** FFA secretion rate was determined in adipose tissue explants of fed and 24 h fasted 14wk HFD-fed mice either in the absence (−Iso) or presence (+Iso) of 1 µM isoproterenol (n = 2–4/group). **D.** mRNA expression of *ghr* was determined by real-time PCR in the epididymal adipose tissue of 14wk-HFD-fed mice (n = 7/group). Each sample was probed three times and gene expression was normalized to *gapdh* and represented as a fold change when compared to control. **E.** Protein content of GHR was determined in protein lysates isolated from epididymal adipose tissue 14wk-HFD-fed control and mGHRKO mice. Liver lysate was used as a positive control (+) (n = 11–15/group, representative blots are shown). β-actin was used to determine equal loading (top). The protein content of GHR was normalized to that of β-actin, and represented as a fold change compared to controls (bottom). **F.** Serum GH levels in the fed state were measured in 14-wk HFD-fed control and mGHRKO mice. Each point on the graph represents an individual mouse (n = 6–8/group). All values are represented as mean ± S.E.M. *− p≤0.05 HFD-fed control versus HFD-fed mGHRKO, #− p≤0.05 mGHRKO/fed versus mGHRKO/fasted, @− p≤0.05 control/fed versus mGHRKO/fed, One-way ANOVA.

Interestingly, the HFD- fed mGHRKO mice had significantly higher serum FFA levels after a 24 h fast **[** **Figure 6A** **]**. We also observed a similar rise in FFA levels in the RC-fed mGHRKO relative to the control mice, suggesting that this adaptation was a consequence of loss of muscle GHR signaling and independent of diet **[Figure S3A]**. Re-feeding for 3 h was associated with significantly greater suppression of lipolysis in the HFD-fed mGHRKO mice, which is in line with their improved insulin sensitivity **[** **Figure 6B** **]**. The lack of an effect of re-feeding on the FFA levels in the HFD-fed control mice was not due to a reduction in food intake or low insulin levels but mainly due to their insulin resistance **[Figure S3B, S3C, S3D]**.

The elevation in FFA levels in the mGHRKO mice at the end of the 24 h fasting period suggested higher FFA flux from the adipose tissue. Thus, we analyzed FFA secretion from adipose tissue explants of fed and fasted HFD-fed control and mGHRKO mice over a period of 3 hours. Adipose tissue explants of 24 h fasted mGHRKO mice demonstrated significantly greater rate of FFA secretion when compared to those from the fed mGHRKO mice; while there was no difference in FFA secretion between the fed and fasted control explants. Interestingly, the FFA secretion rate from the explants of fed mGHRKO mice was significantly lower than that from the fed control mice. However, when lipolysis was stimulated with a β-adrenergic receptor agonist (isoproterenol) all groups, except the fed controls, displayed a significant increase in the rate of FFA secretion, indicating that the mice were responsive to parasympathetic cues **[** **Figure 6C** **]**. Thus, the adipose tissue of the fasted mGHRKO mice has a higher lipolytic rate than those of their fed counterparts.

GH is known to mediate lipolytic effects in the adipose tissue, thus we looked at the expression of GHR in the epididymal adipose tissue, which displays a high rate of lipolysis. Surprisingly, we found a 5- and 1.5- fold increase in mRNA expression and protein content of GHR in adipose tissue lysates of HFD-fed mGHRKO mice, respectively, as compared to the control mice **[** **Figure 6D, 6E** **]**. Further, GH measurements made in the mice in the fed state suggested that the HFD-fed mGHRKO mice may have a trend towards higher GH levels **[** **Figure 6F** **]**. Together these data suggest that GH activity is higher in the HFD-fed mGHRKO mice and this could possibly account for the higher rate of lipolysis in the fasted mGHRKO mice.

## Discussion

We have previously shown that loss of GHR signaling in the skeletal muscle in the mGHRKO mice is associated with a marked protection from the development of HFD-induced insulin resistance, as assessed by several parameters. In addition, the HFD-fed mGHRKO mice had a higher RER in the light phase under conditions of *ad libitum* feeding, indicative of greater whole-body carbohydrate utilization [8]. This indicated that lipid oxidation is suppressed in the mGHRKO mice, and we explored this hypothesis in this study. Our data suggests that basal lipid oxidation is reduced in the skeletal muscle of the mGHRKO mice. However, the induction of lipid oxidation in states of increased energy demand such as fasting and exercise was similar in both the HFD- fed control and mGHRKO mice, suggesting that muscle GHR signaling is not involved in this process. Our results are in line with other studies in GHR knockout (GHRKO) and GH transgenic (GH-Tg) mice, which show an up-regulation of lipid oxidation pathways in response to 30% calorie restriction [14], [15].

PGC1α is a master regulator of lipid oxidation in the skeletal muscle and exerts its effects by associating with several transcription factors. One such transcription factor is ERRα which is an orphan nuclear receptor that is highly expressed in tissues with high energy demand, in a manner similar to PGC1α. Its expression is induced by PGC1α, and it also interacts with PGC1α to activate lipid oxidation [16]. PGC1α also interacts with the PPAR family of transcription factors to regulate lipid metabolism in a tissue-specific manner. In the skeletal muscle, PGC1α/PPAR interaction results in increased FA uptake and activation of lipid oxidation [17], [18]. PPARβ/δ has also been shown to induce PGC1α expression in the muscle [19]. Both ERRα and PPARβ/δ have been shown to induce the expression of PDK4 in a forkhead transcription factor (FoxO1)-dependent manner. PDK4 phosphorylates and inactivates pyruvate dehydrogenase complex resulting in a suppression of glucose oxidation [18], [20]. While we did not find changes in *pgc1α* expression, the expression of *errα*, *pparβ/δ*, and *pdk4* expression were significantly reduced in muscles of the HFD-fed mGHRKO mice, suggesting a suppression of lipid oxidation in the muscles of the HFD-fed mGHRKO mice. Additionally, we observed higher expression of the *mhcIIb* isoform in the quadriceps muscles of the HFD-fed mGHRKO mice, suggesting an increased glycolytic capacity which is also in agreement with the higher RER in the HFD-fed mGHRKO [8].

Any changes in gene expression in the HFD-fed mGHRKO mice could be attributed to their improved insulin sensitivity. We have previously shown that the mGHRKO mice have only a mild improvement in insulin sensitivity under RC-fed conditions [8] **.** Thus, in our initial analysis we included a cohort of RC-fed mice to determine if intrinsic changes exist in the mGHRKO muscles. Indeed, we found a reduction in the expression of *errα* and *pdk4* in the muscles of the RC-fed mGHRKO mice, suggesting that these effects were mediated by the direct loss of GHR signaling in the muscle. It is also likely that the changes in gene expression in the RC-fed mGHRKO mice do not manifest in an overt metabolic phenotype, but in the setting of HFD-induced obesity, lower expression of PGC1α-dependent lipid oxidation genes may in fact be metabolically beneficial. This hypothesis is in line with a report of the lack of an effect of GH treatment on muscle lipid and carbohydrate oxidation in normal, healthy rats [21].

PGC1α has also been shown to regulate mitochondrial biogenesis by the activating the transcription factor NRF1 via direct interaction or indirectly via ERRα [22], [23]. NRF1 activates mitochondrial transcription factors such as TFAM which promote mtDNA replication and stabilization [24]. Despite lower expression of *nrf1* and *tfam* in the HFD-fed mGHRKO mice relative to the controls, we did not detect any changes in mitochondrial content or cytochrome oxidase activity. This observation supports the existence of alternative pathways that could regulate mtDNA replication and maintenance independent of PGC1α. Heterozygous loss of TFAM in mice did not result in a significant reduction in mtDNA copy number in the skeletal muscle, despite lower mtDNA copy number in other tissues like the heart and kidney [25]. Further, PGC1α knockout mice display lower expression of genes involved in mitochondrial biogenesis in the heart and skeletal muscle, but this is not associated with changes in mitochondrial content or the content of oxidative fibers (which are rich in mitochondria) in the heart and skeletal muscle respectively [26]. Moreover, studies in whole-body and muscle-specific PGC1α knockout mice suggest that PGC1α-independent increase in mitochondrial function is possible. The muscle-specific PGC1α knockout mice display compromised mitochondrial structural integrity, which is normalized by voluntary activity [27], [28]. In light of these studies, the higher locomotor activity in the HFD-fed mGHRKO mice may result in activation of alternative pathways of mitochondrial biogenesis and may explain why they do not display alterations in mitochondrial content despite lower expression of *nrf1* and *tfam*.

When challenged with fasting, a condition that activates lipid oxidation, the HFD-fed mGHRKO mice demonstrate increased expression of lipid oxidation genes such as *pgc1α*, *pparα, pparβ/δ,* and *pdk4* to the similar extent as the fasted controls. We next tested our hypothesis by subjecting the mice to a treadmill running challenge, another paradigm that activates lipid oxidation. The HFD-fed mGHRKO mice demonstrated lower RER during the treadmill challenge. We have previously shown that the HFD-fed mGHRKO mice have higher RER in the light phase under standard housing conditions [8]. Thus, taken together our data suggest that while demonstrating reduced lipid oxidation under normal conditions, the HFD-fed mGHRKO are able to adapt to increased energy demand by activating lipid oxidation pathways, a regulation that is essentially lost in obesity and insulin resistance.

The HFD-fed mGHRKO demonstrate up-regulation of *sirt1* expression upon fasting. SIRT1 is a NAD^+^-dependent histone deacetylase that can deacetylate PGC1α and increase its activity [12]. Further, activation of SIRT1 using a synthetic molecule protects mice from diet-induced obesity and increases lipid oxidation in the skeletal muscle by mediating the deacetylation of PGC1α and FOXO1 [29]. Thus, higher *sirt1* expression in the mGHRKO mice upon fasting could result in increased activation of PGC1α in the mGHRKO mice. PGC1α can also be activated by phosphorylation which is mediated by AMPK. However, we did not detect a significant difference in AMPK phosphorylation in the muscles of the control and mGHRKO mice upon fasting. While AMPK is believed to be activated in conditions of low cellular energy, recent studies have in fact reported that AMPK activity is not elevated after a 24 h fast in the skeletal muscle and liver [30].

SIRT3 has been show to regulate global mitochondrial deacetylation; and its expression in the skeletal muscle is down-regulated on HFD feeding, while it is increased in response to exercise and fasting [31], [32]. Further SIRT3 has also been shown to be induced by PGC1α in the ERRα-dependent manner [33]. Fasting induced the expression of *sirt3* only in the HFD- fed mGHRKO mice, but not the control mice. This could either be reflective of their improved insulin sensitivity or greater PGC1α activity (mediated by its deacetylation by SIRT1).

Transgenic over-expression of PGC1α, either in the whole body or specifically in the skeletal muscle, results in increased expression of genes involved in FA uptake in the skeletal muscle [10], [34]. Additionally, we observe a tendency towards increased FAT/CD36 protein content in the mGHRKO, but not control, mice on fasting suggesting greater muscle FA uptake in the fasted mGHRKO mice. Moreover, adipose tissue explants of the fasted mGHRKO mice are significantly more lipolytic than that of the fasted controls. Further, it has been shown that adipose tissue lipolysis and increased delivery of FA to the muscle during exercise occurs before hydrolysis of muscle TG [35], [36]. We also did not observe a significant up-regulation of lipolytic pathways in the muscles of the fasted mGHRKO mice. Thus, we propose that under states of energy demand such as fasting, the adipose tissue of the mGHRKO mice exhibits increased lipolysis and FFA secretion in order to provide substrates for the muscle.

Interestingly, we found higher fed-state circulating GH levels as well as significantly greater adipose tissue GHR expression in the HFD-fed mGHRKO mice. GH mediates adipose tissue lipolysis [37], [38], [39], [40], and this could potentially mediate the increased lipolysis in the fasted mGHRKO mice. There are several factors that could possibly mediate these effects. The higher GH levels in the HFD- fed mGHRKO could be due to their lower insulin levels [8], as insulin has been reported to decrease GH secretion from the anterior pituitary gland [41], [42]. GH has been hypothesized to induce the expression of its receptor, and this may explain the increased expression of GHR in the adipose tissue of the HFD-fed mGHRKO mice (reviewed in [43]). Alternatively, we have also previously reported altered gene expression of myokines, such as myostatin and interleukin-15, in the skeletal muscle of the mGHRKO mice [8], which could potentially also mediate secondary changes in other tissues and may account for the increased GHR expression in the adipose tissue. However, while these are exciting possibilities, further investigation is required to explain these adaptive changes.

In summary, increased skeletal muscle lipid oxidation can have beneficial metabolic effects as seen under exercise-stimulated conditions, or results in metabolic deterioration when activated in the context of obesity and insulin resistance. The predominant difference between the two states is the loss of metabolic flexibility, or the ability of the muscle to shift substrate utilization based on energy demand, under obese, insulin resistant conditions. Our study implicates muscle GHR signaling in regulating basal skeletal muscle lipid oxidation, but not in response to states of increased energy demand such as fasting and exercise.

## Materials and Methods

### Materials

Chemicals and materials were obtained from the following sources: 0.2 micron nitrocellulose membranes, Spectra™ multicolor broad range protein ladder (Thermo Scientific, Rockford, IL); Novex® Tris-Glycine gels, Benchmark™, Novex® Tris-glycine SDS running buffer (10X), Novex® Tris-glycine transfer buffer (25X), Superscript™ II reverse transcriptase, random primers (Invitrogen, Carlsbad, CA); BCA protein assay, bovine albumin (Pierce, Indianapolis, IN); Complete® protease inhibitor cocktail, free fatty acids, half micro test kit (Roche, Indianapolis, IN); sodium flourdie, sodium orthovanadate, tetrasodium pyrophosphate, TritonX-100, pyruvic acid, isoproterenol hydrochloride, anti-growth hormone receptor developed in goat, mouse monoclonal β-actin antibody (clone AC-15), tert-amyl alcohol, 2,2,2 tribromoethanol, cytochrome C from horse heart, catalase from bovine liver, 3,3′-diaminobenzidine tetrahydrochloride (Sigma-Aldrich, Saint Louis, MO); β-glycerophosphate (MP Biochemicals, Solon OH); NaCl, HEPES buffer (1M) (Mediatech, Manassas, VA); CHAPS (Affymetrix, Santa Clara, CA ); fat-free milk powder (Lab Scientific, Livingston, NJ); rat/mouse growth hormone 96-well plate assay, Sensitive rat insulin RIA kit (Millipore, Billerica, MA); dNTP mix (10 mM) (Promega, Madison, WI); RNase- free DNase set, RNeasy® Lipid Tissue Mini Kit, QuantiTect® SYBR® Green PCR kit (Qiagen, Valencia, CA); TG reagent (Pointe Scientific, Canton, MI); IRDye® 800CW conjugated goat polyclonal anti-rabbit IgG, IRDye® 800CW conjugated donkey polyclonal anti-goat IgG, IRDye® 680CW conjugated donkey anti- mouse IgG (Licor Biosceinecs, Lincoln, NE); 50% Glutaraldehyde solution-EM grade (Electron Microscopy Sciences, Hatfield, PA); oligonucleotide primers for gene expression analysis (Eurofins MWG Operon, Huntsville AL); rabbit polyclonal CD36 (H-300), rabbit polyclonal β- tubulin antibody (Santa Cruz Biotechnology, Santa Cruz, CA); TFAM MaxPab rabbit polyclonal antibody D01 (Abnova, Taipei, Taiwan); mouse monoclonal complex IV subunit I (COXI) antibody (MitoSciences, Eugene, OR); rabbit polyclonal phospho-AMPKα^Thr172^ antibody (pAMPK), rabbit polyclonal AMPKα antibody, rabbit polyclonal phospho-HSL^Ser660^ antibody, and rabbit polyclonal phospho-HSL^Ser563^ antibody (Cell Signaling Technology, Danvers, MA); mouse monoclonal lipoprotein lipase (LPL) antibody, and rabbit polyclonal HSL antibody (Abcam Inc, Cambridge, MA). All other common reagents were purchased from Fisher Scientific (Pittsburg, PA) or VWR (Westchester, PA).

### Animals

The generation of the mGHRKO mice has been described previously [8]. Male mice on the C57BL/6 background were used for all experiments, and were housed in the pathogen-free AAALAC accredited animal facilities of the Mount Sinai School of Medicine. All experimental procedures were in accordance with the Institutional Animal Care and Use Committee of the Mount Sinai School of Medicine (IACUC # LA12-00052). The mice were kept on a 12 h light/dark cycle, and had *ad libitum* access to water and either standard mouse chow (LabDiet, Brentwood, MO), or, where indicated, high-fat diet (60% fat calories) (starting from 7 weeks of age) (BioServ, New Brunswick, NJ). In all experiments the mGHRKO (GHR^floxed/floxed/^muscle creatine kinase-Cre^+^) mice were compared to control (GHR^floxed/floxed^) mice. For the 24 h fasting experiments, 14wk-HFD fed mice were separated into 4 groups- control-fed, control-fasted, mGHRKO-fed, mGHRKO-fasted. Mice were anesthesized with Avertin (1∶40 dilution of a 100% solution of 2,2,2 tribromothanol in tert-amyl alcohol), and the tissues were rapidly extracted and snap-frozen in liquid nitrogen. However, prior to freezing the epididymal adipose tissue, a small piece of tissue was taken for the ex vivo lipolysis study (described below).

### Blood Chemistry

Blood glucose levels were measured from the tail using an automated glucometer (Elite; Bayer, Mishawaka, IN). Serum or plasma collected from the tail vein or retro-orbital sinus was used for measurement of circulating insulin, GH and FFA levels as per manufacturers’ instructions.

### Fasting/Re-feeding

At 6:00 p.m of the first day, basal blood glucose levels of the mice were measured from the tail using an automated glucometer (Elite; Bayer, Mishawaka, IN), and the mice were bled from the tail vein for serum and plasma. Subsequently, food was removed from the cage, but the mice had ad libitum access to water. The mice were sampled for blood glucose, serum and plasma at 6:00 p.m the next day, after which a known amount of food (either RC or HFD) was added to the cage. The mice were sampled for blood glucose, serum and plasma again at 9:00 pm; at which time the amount of food remaining in the cage was weighed. Food intake during the re-feeding period was calculated by the formula (gfood_initial_- gfood_final_)/number of mice per cage/(body weight)^0^.^75^/h. The mice were closely monitored for the duration of the study to ensure that they were not adversely affected as a result of the fast. Serum FFA levels and plasma insulin levels were measured from the collected samples as per manufactures’ instructions.

### Ex-vivo Lipolysis

Epididymal adipose tissue was extracted from fed and 24 h fasted mice, rinsed in ice-cold PBS, and minced. 50–70 mg of minced tissue was placed into two wells of a 24-well plate with 1 ml of Krebs-Ringer buffer (calcium chloride 1 mM, magnesium sulfate 1.2 mM, dihydrogen potassium phosphate 1.2 mM, potassium chloride 1.4 mM, sodium chloride 130 mM, pyruvic acid 2 mM, HEPES 20 mM, and bovine serum albumin 2.5%). 1 µM isoproteronol hydrochloride was added into one of the two wells. The plate was incubated at 37°C with mild agitation. 50 µl of buffer was sampled at times 0, 1, 2, and 3 h after isoproteronol addition. FFA levels were measured using 10 µl of sample as per manufacturers’ instructions. FFA secretion rate was determined as the difference in FFA levels in the medium at 0 h and 3 h divided by the duration of the experiment (3 h).

### Indirect Calorimetry

For locomotor acitivity, 14wk-HFD-fed mice were singly housed in metabolic chambers maintained at thermo-neutrality on a 12 h light/dark cycle. Locomotor activity was determined as beam breaks in the horizontal and vertical directions using a CLAMS (Columbus Instruments) open circuit indirect calorimetry system. For the treadmill challenge, 14wk- HFD- fed control and mGHRKO mice underwent two training sessions on the treadmill and on the day of the challenge, the mice were made to run on treadmill, connected to an indirect calorimetry instrument, and the belt speed was incrementally increased every 3 min and the metabolic measurements were made.

### Transmission Electron Microscopy

16wk-old or 14wk-HFD-fed control and mGHRKO mice were anesthetized with Avertin, and were perfused with ice-cold PBS, followed by ice-cold 3% glutaraldehyde. The quadriceps was immediately extracted, minced into 2×2 pieces, fixed in 3% glutaraldehyde, and processed by increased concentrations of ethanol through propylene oxide and embedded in Epon. Thick one micron section were cut and stained with methylene blue and azure II and observed by light microscopy. Representative areas were ultra-thinned and stained with 4% uranyl acetate and Reynold’s lead citrate by the Electron Microscopy Core Lab at the Mount Sinai School of Medicine. Electron micrographs were taken at 5000x magnification using a Hitachi H7650 instrument linked to SIA (Scientific Instruments and Applications) digital camera controlled by Maxim CCD software. 7–10 images from non-overlapping fields were obtained for each animal. ImageJ software (NIH Bethesda, MD) was used to determine the area of individual mitochondria and the mitochondrial density was determined as the ratio of total mitochondrial area to total area of the field. The mitochondrial density for each animal was determined by averaging the mitochondrial density of the different fields.

### Histology and Cytochrome Oxidase Staining

14wk-HFD fed mice were sacrificed and the quadriceps and gastrocnemius were extracted and fixed in 10% PBS-buffered formalin or frozen in liquid-nitrogen cooled isopentane respectively. The quadriceps was subsequently embedded in paraffin, sections were cut, stained with H&E and pictures were obtained using the Olympus AX70 camera (Olympus, Center Valley, PA). NIH-ImageJ software (NIH, Bethesda, MD) was used to quantify the cross-sectional area of at least 170 muscle fibers. Cytochrome oxidase activity in cryosections of the gastrocnemius was assessed by incubating the sections with a buffer containing cytochrome C and catalase. Fibers which were positive for cytochrome oxidase acitivity stained brown due to use of the substrate 3, 3 diaminobenzidine tetrahydrochloride (DAB).

### Western Blot Analysis

The quadriceps and epididymal fat pad were homogenized in protein extraction buffer (50 mM Tris, 150 mM NaCl, 1 mM EDTA, 1.25% CHAPS, 1 mM sodium orthovanadate, 2 mM sodium flourdie, 10 mM sodium pyrophosphate, 8 mM β-glycerophosphate and Complete® Protease Inhibitor Cocktail, pH 7.4), proteins were extracted, and protein concentration was determined using the BCA protein assay using bovine serum albumin as a protein standard. 25–30 µg of proteins were re-suspended in 3x blue loading buffer supplemented with DTT, denatured by boiling at 96°C, and subjected to SDS-PAGE (8–16% acrylamide gradient gels), and transferred to a nitrocellulose membrane. The membranes were blocked in 5% Milk/TBS for an hour at room temperature, incubated with primary antibody diluted in 0.5% Milk/TBS/0.1% Tween-20 overnight at 4°C, and followed by fluorescent secondary antibodies diluted in 0.5%Milk/TBS/0.1% Tween-20 for an hour at room temperature. The signal was developed using the Odyssey Infrared Imaging System (Licor Biosciences, Lincoln, NE). The blots were quantified using the Odyssey Infrared Imaging System Application Software Version 3.0 (Licor Biosciences, Lincoln, NE).

### RNA Analysis

Total RNA from quadriceps and epididymal fat pad was extracted with phenol/chloroform using the RNeasy Lipid Tissue Mini kit the with on-column DNase treatment, and the concentration of RNA was determined using the NanoDrop ND-1000 Spectrophotometer (Thermo Scientific, Wilmington, DE). 1000 ng of RNA was subjected to reverse transcription according to the manufacturer’s instructions (Invitrogen Corp., Carlsbad, CA). The resulting cDNA was diluted to about 200 ul with DNase/RNase- free water, and 3 µl of sample was used per well of a 384-well real-time PCR plate. Real time-PCR was performed using the QuantiTect™ SYBR® green PCR kit (Qiagen, Valencia, CA) in ABI PRISM 7900HT sequence detection systems (Applied Biosystems, Foster City, CA). For each gene, a single sample was assayed 3 times and gene expression was normalized to *gapdh* or *β-actin*. The expression of the MHC isoforms was determined by reverse transcriptase-PCR (RT-PCR) using the following reaction: 95°C –2 min, (94°C –30 sec, 60°C –45 sec, 72°C –1 min)X 25 cycles. *gapdh* was used as an internal control. The band intensity was quantified using ImageJ and the expression of the MHC isoform was normalized to that of *gapdh* and was expressed as a fold change compared to that of the control mice. The primer sequences used were : *pgc-1α* forward: ATGTGTCGCCTTGCTCT and reverse: ATCTACTGCCTGCCCACCTT; *pparβ/δ* forward: AGGCCCGGGAAGAGGAGAAAGAGG and reverse: CGCGTGGACCCCGTA-GTGGA; *pparα* forward: ATGCCAGTACTGCCGTTTTC and reverse: GGCCTT-GACCTTGTTCATGT; *errα* forward: GGAAGTGCTGGTGCTGGGTGT and reverse: GAATTGGCAAGGGCCAGAGCT *pdk4* forward: AAGATGCTCTGCGACCAGTAT and reverse: GAAGGTGTGAAGGAACGTACA *nrf1* forward: GGAGCACTTACTGGAGTCC and reverse: CTGTCCGATATCCTGGTGGT; *tfam* forward: CCTGAGGAAAA-GCAGGCATA and reverse: ATGTCTCCGGATCGTTTCAC; *sirt1* forward: GTAATGT-GAGGAGTCAGCAC and reverse: TTGGACATTACCACGTCTGC; *sirt3* forward: ACTACAGGCCCAATGTCACT and reverse: TTCAACCAGCTTTGAGGCAG; *ghr* forward: GCCTGGGGACAAGTTCTTCTG and reverse: GCAGCTTGTCGTTGGC-TTTCC; *mhcI* forward: CTCCCAAGGAGAGACGACTG and reverse: TTAAGCAGGTCGGCTGAGTT; *mhcIIa* forward: GAACCCTCCCAAGTACGACA and reverse: TAAGGGTTGACGGTGACACA; *mhcIIx* forward: CTTCAACCACCACAT-GTTCG and reverse: GAGCTTCAACCTTGCCTTTG; *mhcIIb* forward: CCAGAGTCACCTTCCAGCTC and reverse: CTTCCCTTTGCTTTTGCTTG; *gapdh* forward: TGAAGGTCGGTGTCAACGGATTTGGC and reverse: CATGTAGGCCAT-GAGGTCCACCAC; *β-actin* forward: ATATCGCTGCGCTGGTCGTC and reverse: AGGATGGCGTGAGGGAGAGC.

### Tissue TG

Total TG were extracted from the liver and gastrocnemius using the chloroform-methanol method [44]. Briefly, approximately 100 mg of gastrocnemius tissue was crushed on dry ice using a mortar and pestle and transferred to a glass tube. The tissues were incubated in 3 mL of 2∶1 chloroform: methanol mixture for 4 h with agitation, after which 1.5 mL of 0.1 M NaCl was added to the tubes, mixed by vortexing and centrifuged at 1000 rpm for 10 min at room temperature to separate the organic and aqueous phases. The lower organic phase containing the TG was transferred to a new glass tube using a glass Pasteur pipette and the liquid was evaporated under a steady stream of nitrogen gas. The remaining TG pellet was re-suspended in 200 uL of a 3 M potassium hydroxide in 65% ethanol solution and incubated at 70°C for 60 min in a water bath, and stored at −20°C. The samples were diluted 1∶10 in water and TG content was quantified using the TG reagent.

### Statistical Analysis

All data are represented as mean ± S.E.M. One-way (for comparison of two groups) or two-way (for comparison of three or more groups) ANOVA with Holm-Sidak post-hoc test was performed using SigmaStat for Windows (version 3.5; Systat Software, Inc., Chicago, IL).

## Supporting Information

Figure S1 **Analysis of fiber type in the mGHRKO mice. (A,C).** Average muscle cross sectional area in about 175 fibers was measured using ImageJ software in RC-fed **(A)** and HFD-fed **(C)** mice (n = 6–7/genotype/diet). **(B,D)** mRNA expression of myosin heavy chain (MHC) isoforms as measured by RT-PCR in quadriceps muscles of control and mGHRKO mice under RC-fed **(B)** and HFD-fed **(D)** conditions. Band intensity was quantified using ImageJ software and represented as a fold change compared to control mice (n = 6–14/genotype/diet). **E.** Muscle cytochrome oxidase acitivity in gastrocnemius muscle of HFD-fed control and mGHRKO mice. Scale bar represent 500 µm (n = 4/genotype, representative images are shown). All values are represented as mean ± S.E.M. *− p≤0.05 control versus mGHRKO.
(TIF)

Figure S2 **Basal expression of sirtuins.** mRNA expression of *sirt1* and *sirt3* as measured by realtime PCR analysis in quadriceps muscles of control and mGHRKO mice under HFD-fed conditions (n = 7–8/genotype). All values are represented as mean ± S.E.M.
(TIF)

Figure S3 **Response of mice to a fasting/re-feeding challenge.** **A.** FFA levels were determined in the serum of 16 week old RC-fed mice at baseline (0), after 24 h fasting (24), and after 3 h of re-feeding (+3). **B.** Blood glucose levels was measured in whole blood of mice at baseline (0), after 24 h fasting (24), and after 3 h of re-feeding (+3). **C.** Food intake during the re-feeding phase was determined from the difference in weight of food added at the start and end of the re-feeding phase. **D.** Insulin levels was determined in plasma of the mice at the indicated times (n = 4–11/genotype/diet). All values are represented as mean ± S.E.M. a- p≤0.05 HFD-fed control versus HFD-fed mGHRKO, b- p≤0.05 RC-fed control versus RC-fed mGHRKO, c- p≤0.05 RC-fed control versus HFD-fed control, d- p≤0.05 RC-fed mGHRKO versus HFD-fed mGHRKO. Two-way ANOVA.
(TIF)
