# Supplementary figures and images for: Skeletal Muscle Growth Hormone Receptor Signaling Regulates Basal, but Not Fasting-Induced, Lipid Oxidation
Source: PLoS One. 2012 Sep 14;7(9):e44777. doi: 10.1371/journal.pone.0044777 (PMC3443095; doi:10.1371/journal.pone.0044777)

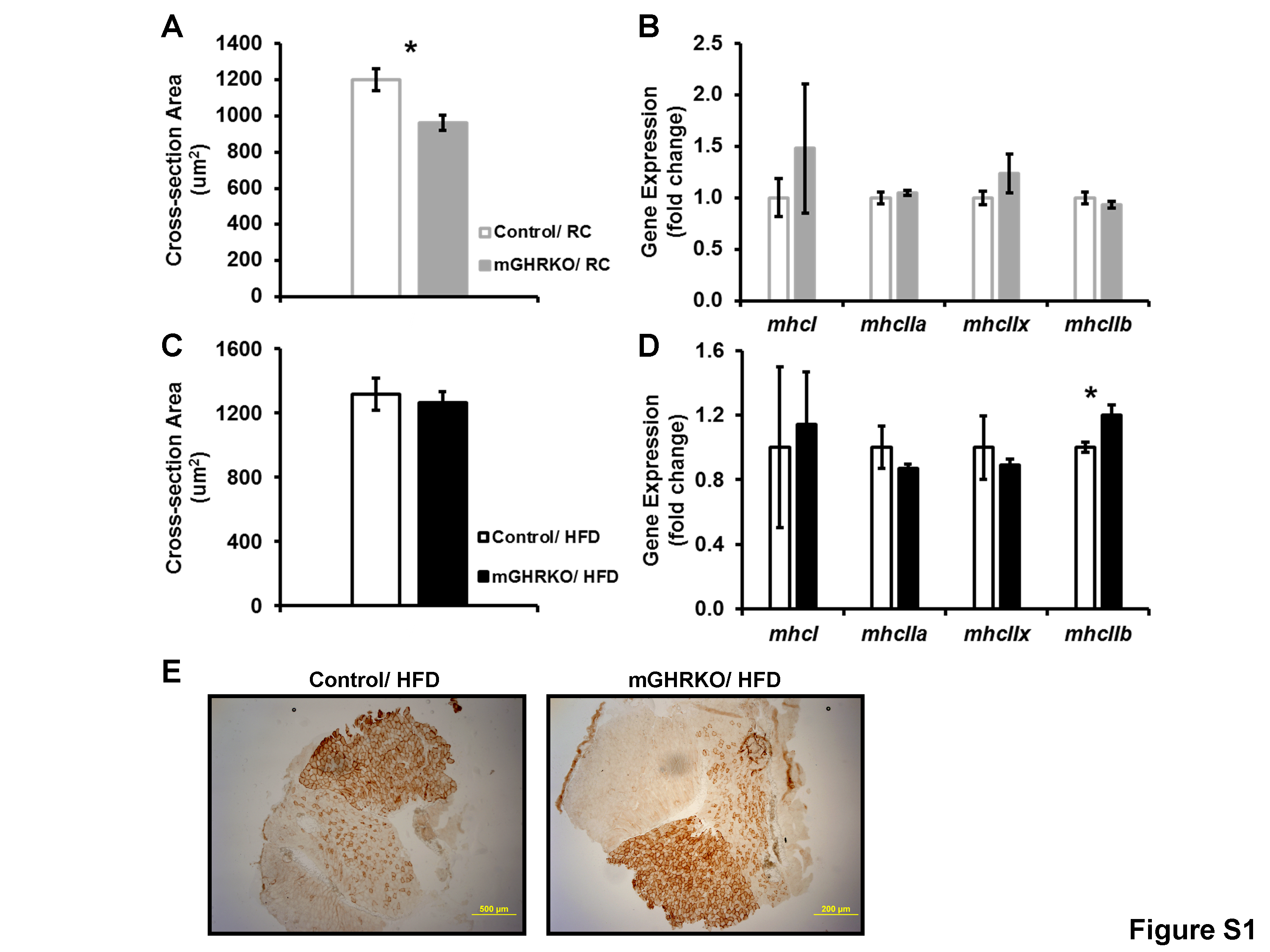

Supplement: Figure S1 — Analysis of fiber type in the mGHRKO mice. (A,C). Average muscle cross sectional area in about 175 fibers was measured using ImageJ software in RC-fed (A) and HFD-fed (C) mice (n = 6–7/genotype/diet). (B,D) mRNA expression of myosin heavy chain (MHC) isoforms as measured by RT-PCR in quadriceps muscles of control and mGHRKO mice under RC-fed (B) and HFD-fed (D) conditions. Band intensity was quantified using ImageJ software and represented as a fold change compared to control mice (n = 6–14/genotype/diet). E. Muscle cytochrome oxidase acitivity in gastrocnemius muscle of HFD-fed control and mGHRKO mice. Scale bar represent 500 µm (n = 4/genotype, representative images are shown). All values are represented as mean ± S.E.M. *− p≤0.05 control versus mGHRKO. (TIF) [file pone.0044777.s001.tif]

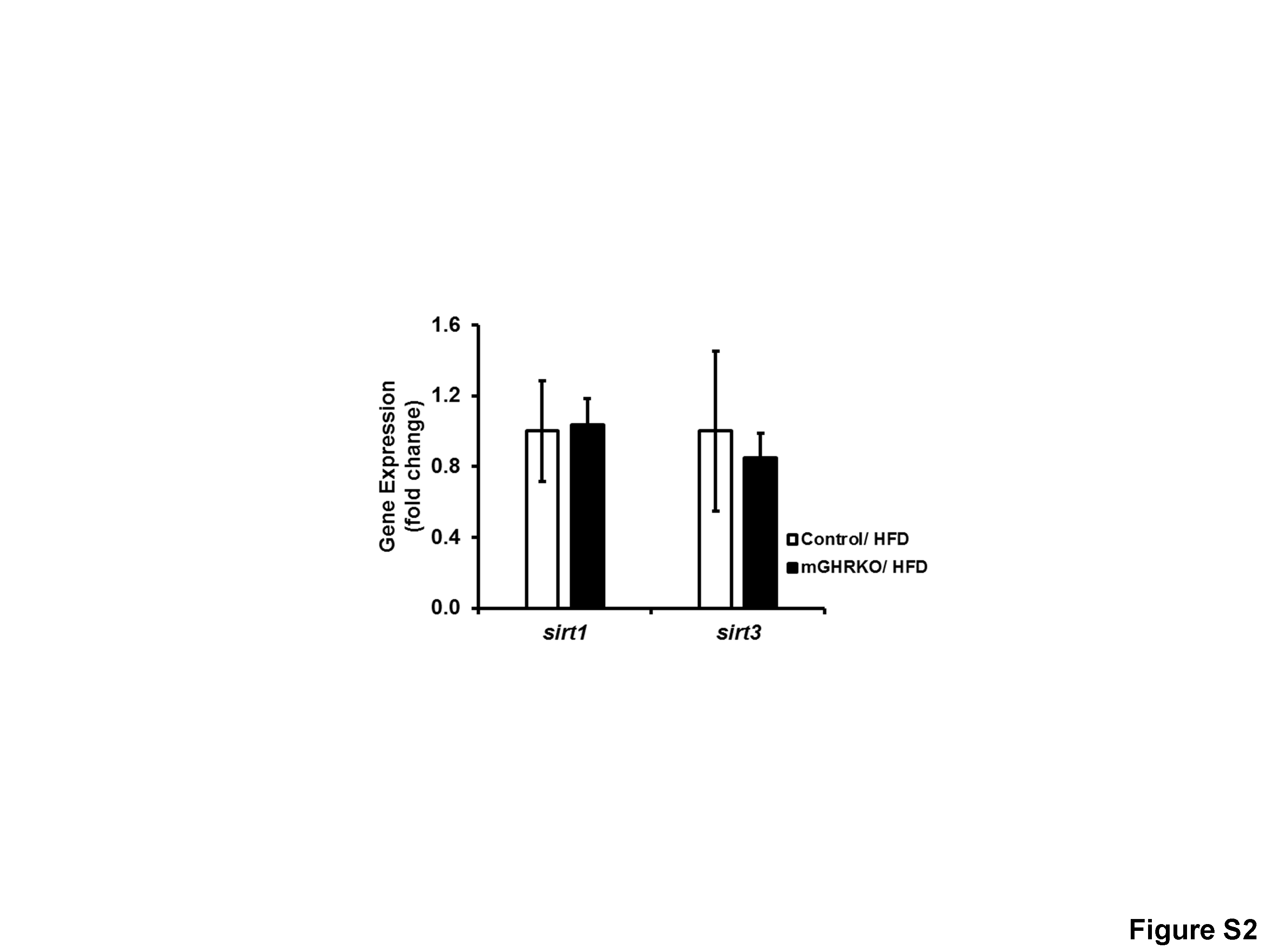

Supplement: Figure S2 — Basal expression of sirtuins. mRNA expression of sirt1 and sirt3 as measured by realtime PCR analysis in quadriceps muscles of control and mGHRKO mice under HFD-fed conditions (n = 7–8/genotype). All values are represented as mean ± S.E.M. (TIF) [file pone.0044777.s002.tif]

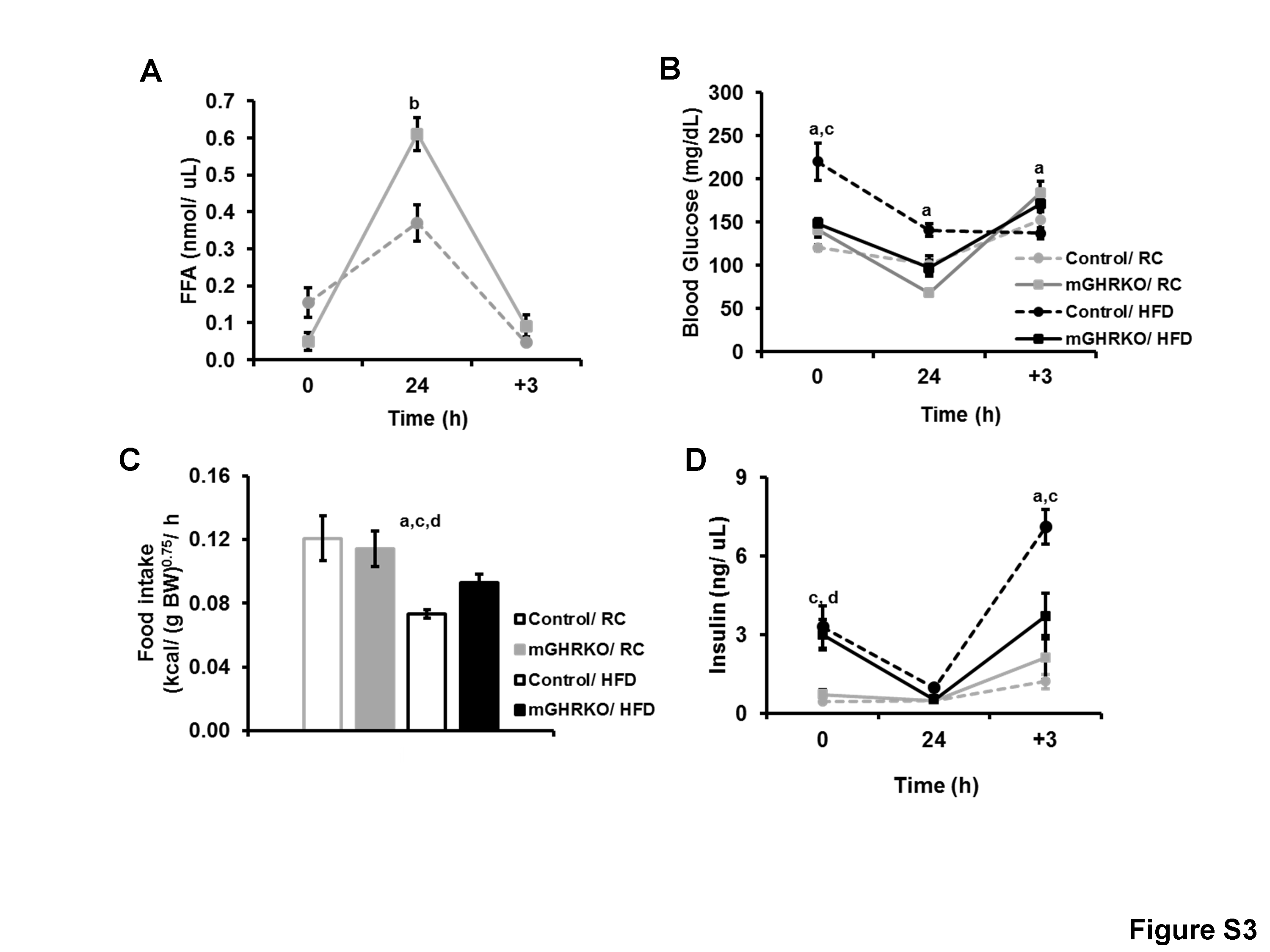

Supplement: Figure S3 — Response of mice to a fasting/re-feeding challenge. A. FFA levels were determined in the serum of 16 week old RC-fed mice at baseline (0), after 24 h fasting (24), and after 3 h of re-feeding (+3). B. Blood glucose levels was measured in whole blood of mice at baseline (0), after 24 h fasting (24), and after 3 h of re-feeding (+3). C. Food intake during the re-feeding phase was determined from the difference in weight of food added at the start and end of the re-feeding phase. D. Insulin levels was determined in plasma of the mice at the indicated times (n = 4–11/genotype/diet). All values are represented as mean ± S.E.M. a- p≤0.05 HFD-fed control versus HFD-fed mGHRKO, b- p≤0.05 RC-fed control versus RC-fed mGHRKO, c- p≤0.05 RC-fed control versus HFD-fed control, d- p≤0.05 RC-fed mGHRKO versus HFD-fed mGHRKO. Two-way ANOVA. (TIF) [file pone.0044777.s003.tif]
